# Whole mitochondrial DNA sequencing in Alpine populations and the genetic history of the Neolithic Tyrolean Iceman

**DOI:** 10.1038/srep18932

**Published:** 2016-01-14

**Authors:** V. Coia, G. Cipollini, P. Anagnostou, F. Maixner, C. Battaggia, F. Brisighelli, A Gómez-Carballa, G. Destro Bisol, A. Salas, A. Zink

**Affiliations:** 1 Accademia Europea di Bolzano (EURAC-Research), Istituto per le mummie e l´Iceman, Bolzano, Italy; 2 Dipartimento Biologia Ambientale, Università La Sapienza, Roma, Italy; 3 Department of Zoology, University of Oxford, South Parks Road, Oxford, OX1 3PS, UK; 4 Unidade de Xenética, Departamento de Anatomía Patolóxica e Ciencias Forenses, and Instituto de Ciencias Forenses, Facultade de Medicina, Universidade de Santiago de Compostela, Galicia, Spain; 5 Istituto Italiano di Antropologia, Roma, Italy

## Abstract

The Tyrolean Iceman is an extraordinarily well-preserved natural mummy that lived south of the Alpine ridge ~5,200 years before present (ybp), during the Copper Age. Despite studies that have investigated his genetic profile, the relation of the Iceman´s maternal lineage with present-day mitochondrial variation remains elusive. Studies of the Iceman have shown that his mitochondrial DNA (mtDNA) belongs to a novel lineage of haplogroup K1 (K1f) not found in extant populations. We analyzed the complete mtDNA sequences of 42 haplogroup K bearing individuals from populations of the Eastern Italian Alps – putatively in genetic continuity with the Tyrolean Iceman—and compared his mitogenome with a large dataset of worldwide K1 sequences. Our results allow a re-definition of the K1 phylogeny, and indicate that the K1f haplogroup is absent or rare in present-day populations. We suggest that mtDNA Iceman´s lineage could have disappeared during demographic events starting in Europe from ~5,000 ybp. Based on the comparison of our results with published data, we propose a scenario that could explain the apparent contrast between the phylogeographic features of maternal and paternal lineages of the Tyrolean Iceman within the context of the demographic dynamics happening in Europe from 8,000 ybp.

The Tyrolean Iceman (also known as Ötzi or Similaun Man) was discovered in 1991 on the *Tisenjoch* Pass in the Italian part of the Ötztal Alps, close to the Austrian border (3210 m s.l.m.). The Iceman is one of the oldest natural European mummies (dated to 5350–5100 ybp), and an extraordinarily well-preserved individual due to a spontaneous freeze-drying process. Scientific interest is further increased by the preservation of parts of his clothes and equipment (e.g. loincloth, shoes, leggings, axe, bow).

This important finding provided unprecedented insights into aspects of the daily life during the Copper Age in central Europe^1^,^2^,^3^. Both stable isotopes analyses of Iceman’ s teeth and bones and the identification of pollen and moss fragments in his intestines have pinpointed his origin to south of the Alpine ridge to a few valleys within ~60 kilometers South-East of the discovery site (*Vinschgau* and *Schnals* valleys in actual South-Tyrol, Eastern Italian Alps)^4^,^5^.

The Tyrolean Iceman has also been extensively characterized genetically. Whole genome sequencing data indicate that the Iceman was blood group 0, lactose intolerant and genetically predisposed to cardiovascular diseases^6^. Moreover, Keller and colleagues^6^ showed that the Iceman belongs to the Y-chromosome haplogroup G2a-L91. Comparing the Iceman’s genomic data to the human reference genome (UCSC hg19) specified the Iceman´s lineage into one of the four sub-branches of G2a L-91 (G2a2a1a2a1a-FGC5672) following the latest version of the Y-DNA Haplogroup Tree^7^. Comparative analysis based on informative autosomal SNPs and Y-chromosome data showed a clear link with genetic diversity of extant populations further supported by recent whole genome-data analysis^8^. However, an important question regarding the Iceman genetic heritage remains unanswered. In fact, despite the mitochondrial DNA (mtDNA) of the Iceman being well characterised^9^,^10^,^11^,^12^, the relation of his lineage with present-day mitochondrial diversity remains elusive. The first study on the complete mtDNA of the Iceman^11^ showed that the ancient mitogenome was a novel lineage of mtDNA haplogroup K1 (named K1f), not yet detected in extant populations. Ermini *et al.*^11^ proposed that the Iceman’s haplogroup may remain undetected simply due to inadequate sampling. This hypothesis should be considered due to the paucity of data regarding K1 haplogroups available at the time of the study, especially from alpine populations, which are putatively in genetic continuity with the Iceman. Alternatively, the K1f haplogroup and its closest neighbors may have been lost during repeated demographic and population events occurring in the Alps since the Neolithic.

To shed new light on these issues, we analyzed the mitogenomes of 42 K–haplogroup samples from Eastern Italian Alps and compared the Iceman´s sequence with a large dataset of worldwide K1 mitogenomes. The results are discussed with currently available ancient mtDNA data from European Neolithic specimens. Finally, we combine mtDNA and Y-chromosomal data to propose a scenario that could explain the complex genetic history of the Tyrolean Iceman.

## Results and Discussion

### The Iceman mitochondrial genetic heritage

**Figure 1:**
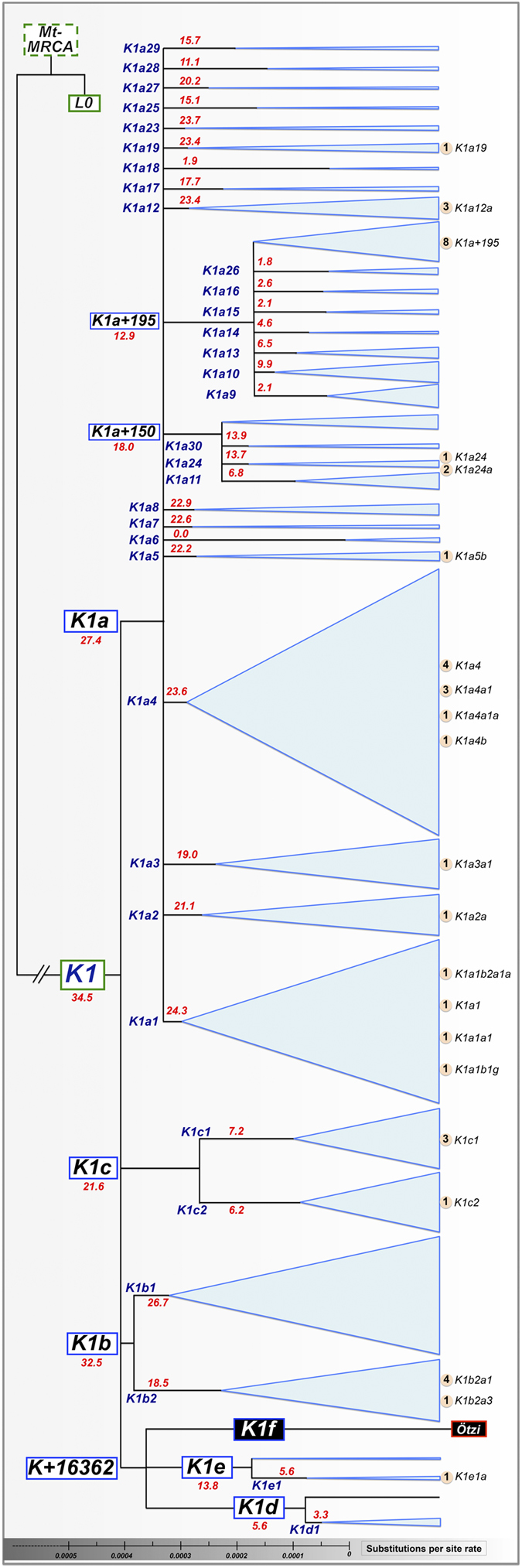
Main skeleton of haplogroup K1 and its branches based on all of the mitogenomes. The area of the triangles is proportional to the occurrence of K1 haplogroups in the total dataset (see also Table S2). The haplogroup assignment of K1 Alpine mitogenomes (thirty-six from the present study and six from^37^) are listed on the right-hand side of the tree. Age estimates of haplogroups are reported close to each branch (see also Table S3).

The mitogenomes of 42 haplogroup K individuals from Eastern Italian Alps were analyzed (Table S1). The most frequently observed K1 Alpine haplogroups were K1a4 and K1a1, similar to the worldwide K1 distribution (Fig. 1) and K1a + 195 (Table S2). Rare haplogroups were also observed in this study such as K1a19 and K1a24, and K1e1, which was represented in our dataset by a novel haplotype defined by a specific transition in the coding region (transition A15244G; sample #111Gm in the Fig. S2). Neither the Iceman’s lineage nor any other intermediate haplotypes of K1f were detected.

**Figure 2:**
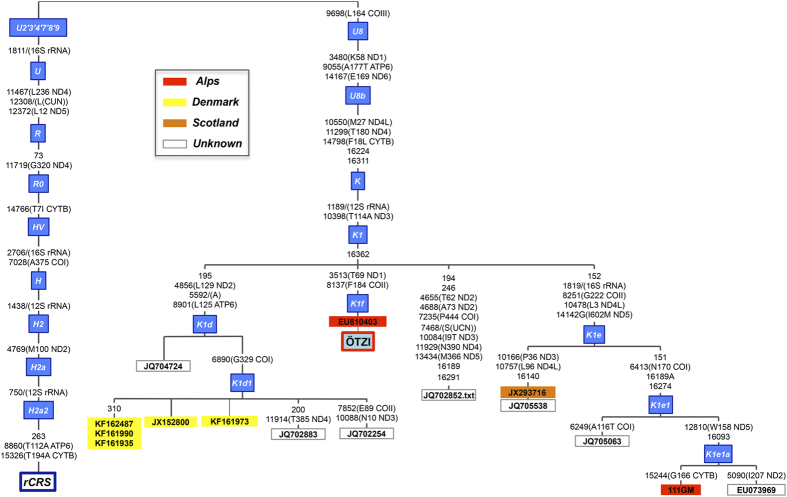
Most parsimonious phylogenetic tree of the K1 + 16362 cluster.

Next, we analyzed all K1 mitogenomes available in the literature, scanning databases, in combination with our dataset (*n* = 1077; Table S2). The whole K1 tree was updated and the Iceman’s sequence was added to the phylogeny (Fig. 1). This tree was compared with the one presented by Ermini and collaborators^11^ that used in total 115 K sequences (85 of which were K1 mitogenomes). The Iceman’s mitogenome falls within a cluster defined by transition T16362C (K1 + 16362) that includes K1d (5.6 thousand years [kya]; standard error: 0.6–10.7) and K1e (13.8 kya; standard error: 6.5–21.4) haplogroups. The collected data created a clearer and more robust representation of K1 + 16362 cluster despite being defined exclusively by an unstable mutation on the rapidly mutating mtDNA control region. Indeed, only one sequence of unknown origin (EU073969) shared the mutation T16362C with the Iceman´s mitogenome in the tree built by Ermini and colleagues^11^. Currently, this cluster encompasses 13 other modern sequences mostly derived from individuals of known European ancestry (five sampled in Denmark, one in Scotland, one in the Alps, and six of unknown origin; see Fig. 2). The closest observed haplotype (JQ704724) to the Iceman’s mitogenome is, however, quite distant from K1f, being separated by six mutational steps (five of which are in the coding region).

Overall, the results indicate that K1f has been lost from present-day populations, rather than being a rare haplotype undetected due to inadequate sampling. With a binomial distribution^13^,^14^ given the size of our dataset, we estimated (with a 95% of probability) that the K1f lineage should not occur with a frequency above 0.3%. In addition, the most parsimonious phylogenetic tree showed that there exist no intermediate haplotypes connecting the Tyrolean Iceman’s lineage to the main root of K1 (Fig. 2).

Recently, considerable knowledge has been gained on the mtDNA diversity of ancient Europeans^15^. It appears that the Iceman´s haplogroup may not be the only case of a described mtDNA lineages that has apparently been lost. Two examples include lineages from haplogroup H (H46b and H88) that have been detected in two Linear Pottery culture individuals (LBK, 5450-4775 BC) in Germany^16^. However, H46b and H88 haplogroups are only one-step mutation away from the extant ancestral haplogroups (H46 and H*, respectively). This is in contrast to the described phylogeographic features of the K1f haplogroup, suggesting that the entire branch of K1f was lost.

### Reconstructing the genetic history of the Tyrolean Iceman

The focused analysis of the Tyrolean Iceman has produced important information on his origin and genetic relations between Iceman and extant European populations. The availability of data on genetic markers with different modes of inheritance and rates of evolution allows to perform comparative analysis that shed further light on the genetic history of the Tyrolean Iceman. Interestingly, there is a contrast between the Iceman´s maternal and paternal genetic heritage. While the branch of mtDNA K1f lineage has probably been lost from present-day populations according to this study, the Y-chromosome branch G2a-L91 is still observed in Europe, and reaches remarkable frequencies ( > 10%) in groups from the Mediterranean area, i.e. Sardinians and Corsicans^6^,^17^. How can this pattern be explained?

The direct comparison of the evolutionary history of specific female and male lineages can be complicated by differences in their phylogenetic structure and resolution. However, it is likely that the dissimilarity observed in the diachronic patterns of the Iceman’s genetic lineages are due mainly to the distributions of K1f and G2a in Europe prior to 5,000 ybp and later demographic events that shaped the European genetic structure.

**Figure 3:**
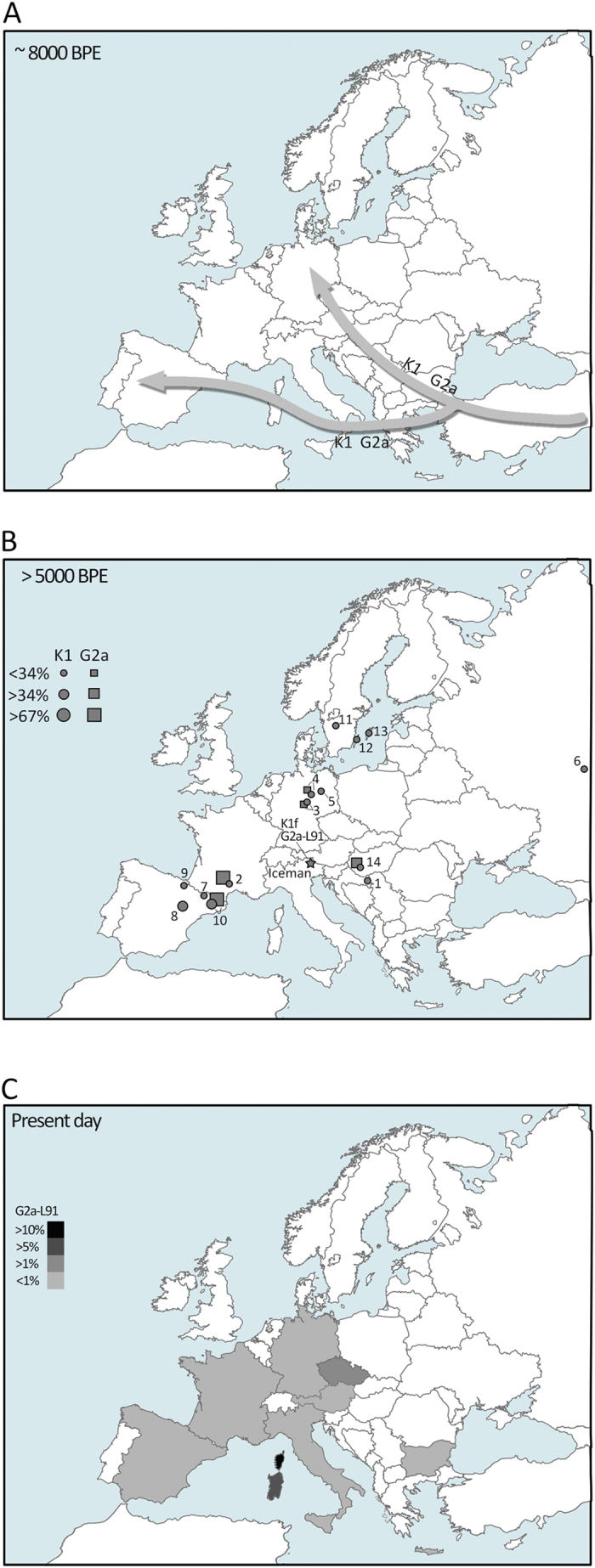
(**A**) Migrations occurred around 8,000 ybp of Early Neolithic people from the Near East to Europe carrying the main mtDNA (K1) and Y chromosome (G2a) haplogroups according to recent data on ancient DNA studies^15^,^18^. (**B**) Distribution of mtDNA K1 (circles) and Y chromosome G2a (rectangles) haplogroups in ancient samples dated > 5,000 ybp and their absolute frequencies. Site 1: *Vinkovci Nama*, Croatia^23^; site 2: *Treilles at Saint-Jean-et-Saint-Paul*, France^19^; site 3: *Derenburg-Meerenstieg II*, Germany^22^; site 4: *Halberstadt-Sonntagsfeld*, Germany^21^; site 5: South of *Saxony-Anhalt*, Germany^18^; site 6: *Yamnaya*, Russia^21^; site 7: *Els Trocs*, Spain^21^; site 8: *La Mina*, Spain^21^; site 9: *Navarre*, Spain^38^; site 10: *Avellaner*, Catalonia, Spain^20^; site 11: *Gökhem*, Sweden^39^,^40^; site 12: *Köpingsvik*, Sweden^40^; site 13: *Gotland*, Sweden^40^; site 14: *Alsónyék-Bátaszék, Mérnöki telep, Lánycsók, Gata-Csotola, Bölcske-Gyűrűsvölgy, Budakeszi, Szőlőskert-Tangazdaság*, Hungary^23^. (**C**). Distribution and approximate frequencies of haplogroup G2a-L91 in Europe based on the data from Rootsi *et al.*^17^. Maps of Europe available from Wikipedia Common web page (https://commons.wikimedia.org/wiki/File:Blank_political_map_Europe_in_2006_WF.svg?uselang=it#filelinks) were modified using Adobe Photoshop CS6 software.

Recent ancient DNA studies suggest that both mtDNA haplogroup K1 and Y-chromosome G2a reached the European continent around 8,000 ybp by migrations of Early Neolithic people from the Near East through continental and Mediterranean routes^15^,^18^,^19^,^20^ (see Fig. 3A). Subsequently, these haplogroups spread across the continent, as shown by the distribution of K1 and G2a from several Europeans specimens dated > 5,000 ybp (see Fig. 3B and its legend for references and details). At that time, the K1 haplogroup was present in West, South, East, and North Europe with its main branches (K1a, K1b) and rarer branch like K1e, whereas K1f was virtually restricted to the Alps since it has not been observed outside this region. On the other hand, G2a was the predominant Y-chromosome lineage observed in the European Neolithic samples analyzed thus far^15^,^19^,^20^,^21^,^22^,^23^.

While the Iceman’s Y-chromosome lineage was part of the Early Neolithic genetic substrate, as suggested from current distributions of G2a-L91 (Fig. 3C), the mtDNA K1f lineage developed locally in the Eastern Alps at least from ~5,200 ybp (the time of the Iceman).

After 5,000 ybp, population expansion, migrations and admixture occurred throughout the continent, re-shaping the genetic structure of Europeans^8^,^15^,^21^. These events led to the almost complete replacement of the Y-chromosome haplogroup G2a-L91 by other haplogroups (e.g. R1b). However, Sardinians and Corsicans maintained such genetic feature due to geographic isolation, as supported from whole-genome data^6^,^8^ (Fig. 3). Conversely, the K1f branch may have been completely replaced by haplogroups that are frequent today (e.g haplogroup H). This process was probably favored by the stationary demographic state of population groups bearing K1 haplotypes in the Alps as suggested by the Bayesian Skyline Plot (BSP) of Fig. S3. This is additionally supported by archaeological data^24^ that suggests low population density during the Neolithic and the Copper Age (5,450-4,150 ybp) in the Iceman’s territory (*Vinschgau* valley), while significant demographic growth started ~2,000 years later during the Middle Bronze Age.

In conclusion, by combining genetic data from modern-day populations and European Neolithic specimens, we gained new insights into the genetic history of the Tyrolean Iceman. This study demonstrates the continued importance of this unique specimen to shed light on the early migrations in Europe, and marks a first step towards a systematic study of the genetic history of ancient Alpine groups.

## Methods

### Laboratory analysis

Thirty-nine DNA samples belonging to the haplogroup K were selected from more than 800 individuals from the Eastern Italian Alps (from Trentino-Alto Adige, Veneto and Friuli regions) analyzed at low resolution in previous studies^25^,^26^,^27^,^28^ (Fig. S1). Additionally, forty-three new buccal swabs samples from South-Tyrol were collected after the authorization by the Ethic Committee of *Azienda sanitaria dell’Alto Adige.* Informed consent was obtained from all subjects. The experimental protocol was carried out in accordance with the approved guidelines and was approved by the above-mentioned Ethic Committee.

DNA extraction was carried out using a commercial kit (Gentra Puregene Buccal Cell Kit, Qiagen). Then, samples were typed for informative SNPs of the mtDNA haplogroup K status (A10550G, T11299C and T14798C of the coding region) by using the SNAPShot method (Applied Biosystems Carlsbad, CA). Only three samples (~7%) carried the diagnostic variants. Afterwards, the total K samples (*n* = 42) were analyzed for the whole mtDNA genome (16569 base pairs) using the methods described by Torroni and collaborators^29^ with minor modifications. Nomenclature of mtDNA variants were referred against the rCRS^30^.

In order to classify the sequences in the sub-lineages of haplogroup K, we compared our data with the worldwide human mtDNA phylogenetic tree (PhyloTree Build 16)^31^ using Haplogrep 2.0 Beta version (http://haplogrep.uibk.ac.at/blog/visuali ze-yo/) and supervised manually. The new sequences were deposit in GenBank (accession numbers from KT749793 to KT749816).

### Statistical analysis

We collated from different sources (GeneBank, The 1000 Genomes Project) all available complete K1 mitogenomes from present-day populations (*n* = 1042; October 2014). The 1000 Genome Project mitogenomes were processed using in-house scripts^32^. We also compiled data regarding the geographic origin of the samples. More than four hundred mitogenomes (*n* = 409) were from individuals with reported European ancestry, 440 with unknown geographic origin and the remaining from non-European regions (see Table S2 for more details).

Phylotree Build 16^31^ was used as a reference to draft the skeleton of K1 haplogroup and its branches; some branches were updated according to new available data (Fig. 1). For the most closely related sequences of the Iceman haplotype we reconstructed the phylogeny using the reduced-median algorithm^33^ (Fig. 2). Reticulations were resolved using the relative mutation rates at each position^34^. The phylogenetic tree was drafted using mtPhyl v3.520 (http://eltsov.org) and adjusted manually according to Phylotree Built 16^31^ and the data added in the present study.

We estimated the Time of the Most Recent Common Ancestor (TMRCA) of clades utilizing a time-dependent clock incorporating a correction for purifying selection^34^ and using the maximum likelihood (ML) procedure with PAML 3.13^35^. A skeleton of the tree based on a well known mtDNA phylogeny (Phylotree Build 16) was initially provided to the software in newick format. Then, TMRCA was obtained assuming the HKY85 mutation model (excluding indels, and hotspot mutations such as 16182C, 16183C and 16519C), and used the gamma-distributed rates (approximated by a discrete distribution with 32 categories) and three partitions: Hypervariable region I (HVS-I: positions 16051–16400), Hypervariable region II (HVS-II: positions 68–263), and the coding region. A set of outgroups from haplogroups were used that represent most of the main branches of the worldwide phylogeny, from the rCRS to the oldest African nodes^31^.

The BSP showing changes of the effective population size of haplogroup K1 through time was performed with the Bayesian Evolutionary Analysis Sampling Trees (BEAST)^36^ software. The analysis was made using only the mtDNA coding region. MCMC samples were based on 40,000,000 generations with the first 4,000,000 generations discarded as burn-in. We used a HKY substitution model and a strict clock with a substitution rate of 1.69 × 10^-8^ as reported in^34^. BEAST outputs were analyzed with the software Tracer (http://tree.bio.ed.ac.uk/software/tracer/).

## Additional Information

**How to cite this article**: Coia, V. *et al.* Whole mitochondrial DNA sequencing in Alpine populations and the genetic history of the Neolithic Tyrolean Iceman. *Sci. Rep.* **6**, 18932; doi: 10.1038/srep18932 (2016).

## Supplementary Material

Supplementary Information

Supplementary Table S1

Supplementary Table S2

Supplementary Table S3
