# Supplementary material for: Whole mitochondrial DNA sequencing in Alpine populations and the genetic history of the Neolithic Tyrolean Iceman
Source: Sci Rep. 2016 Jan 14;6:18932. doi: 10.1038/srep18932 (PMC4725900; doi:10.1038/srep18932)

Whole mitochondrial DNA sequencing in Alpine populations and the genetic history of the Neolithic Tyrolean Iceman

Coia Valentina, Cipollini Giovanna, Anagnostou Paolo, Maixner Frank, Battaggia Cinzia, Brisighelli Francesca, Gómez-Carballa Alberto, Destro Bisol Giovanni, Salas Antonio, Zink Albert

Contents

Figure S1. Approximate places of origin of the K alpine samples (yellow stars) analyzed in this study and place of discovery of the Tyrolean Iceman (red star). Map was created by Kathrin Renner (Eurac-Research) using ESRI 2011 ArcGIS Desktop: Release 10.

Figure S2. Median Joining network of 42 K sequences from alpine populations analyzed in this study more 6 K sequences from south Tyrolean area[1] (see also Table S2). Weight at the different positions were assigned according to[2].

Figure S3. Bayesian skyline plot showing changes thought the time (kya, abscissa) of the effective population size (Ne, ordinate) of haplogroup K1 in the Alps.

Table S1. Total K Alpine samples analyzed in this study. This table is provided in a separate file.

Table S2. Total K1 mitogenomes used in this study. This table is provided in a separate file.

Table S3. TMCRA estimates of K1 haplogroup and ist sub-clades. This table is provided in a separate file.

References

1. Pichler, I. *et al.* Drawing the history of the Hutterite population on a genetic landscape: inference from Y-chromosome and mtDNA genotypes. *Eur. J. Hum.Genet*. **18,** 463–470 (2010).
2. Brandstätter, A. *et al.* Timing and deciphering mitochondrial DNA macro-haplogroup R0 variability in Central Europe and Middle East. *BMC Evol. Biol*. **8,** 191 (2008).

Figure S1

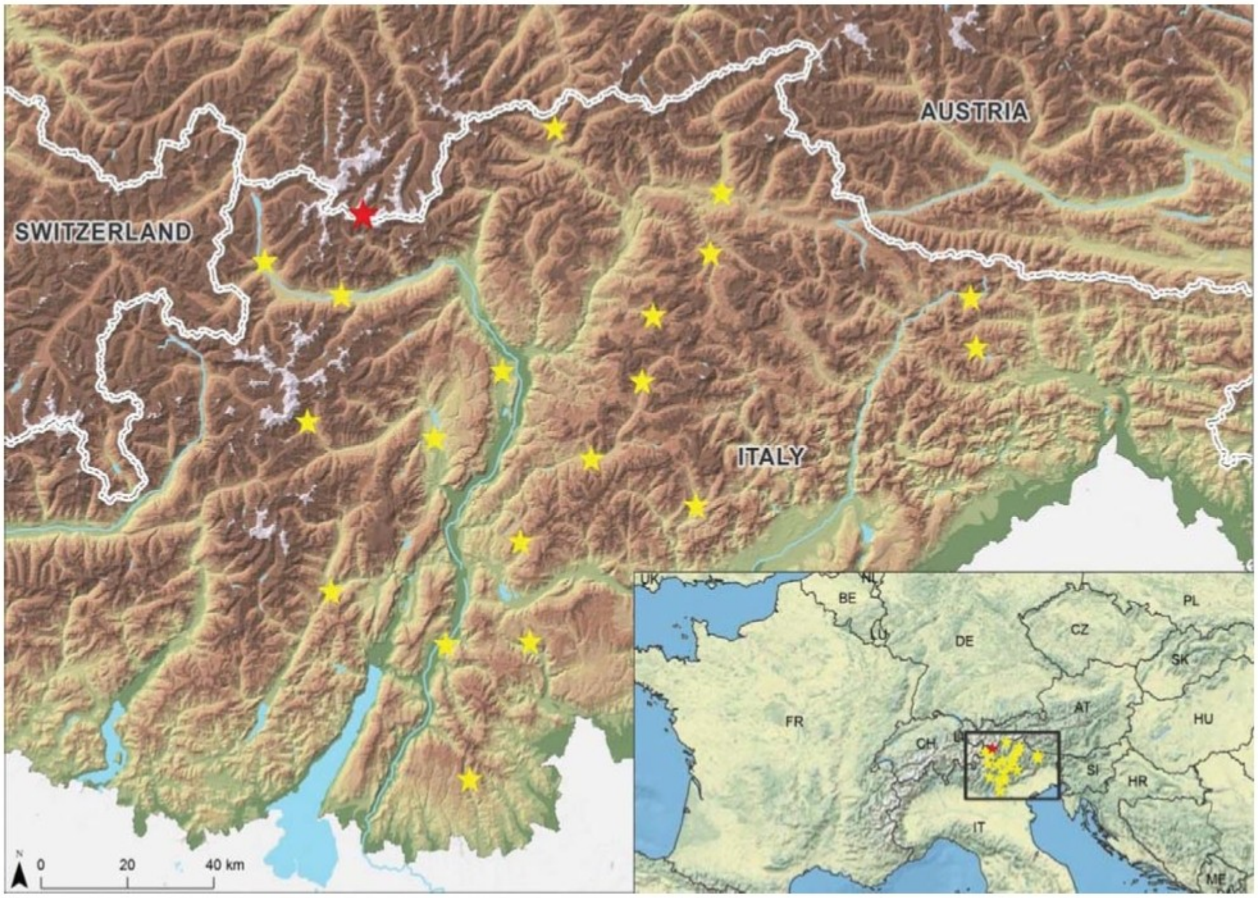
AUSTRIA
SWITZERLAND
ITALY
N
0
20
40 km
UK
NL
BE
DE
CZ
PL
SK
FR
CH
AT
HU
SI
HR
IT
ME

Figure S2

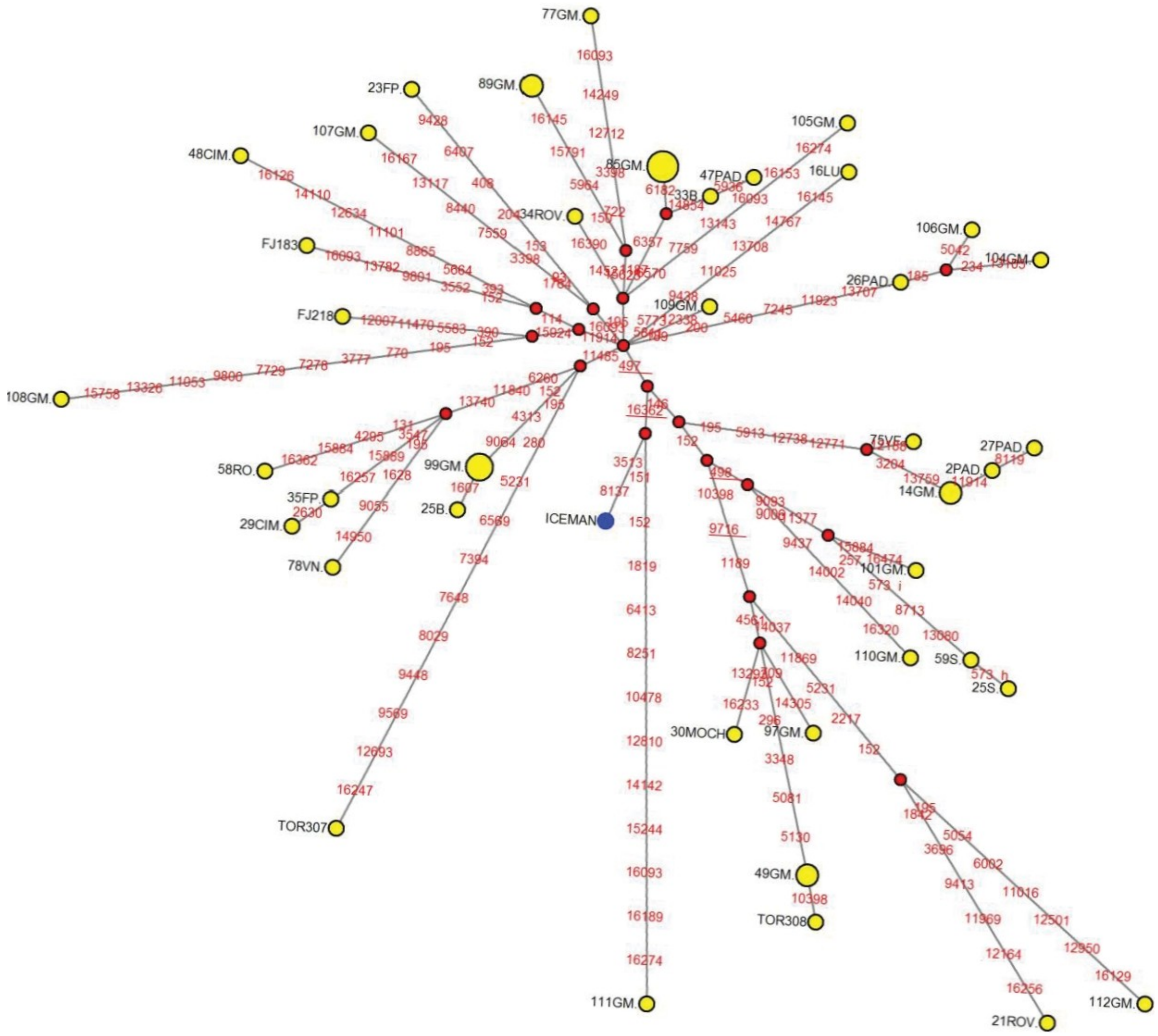

77GM.
23FP.
89GM.
105GM.
107GM.
48CIM.
85GM.
47PAD.
16LU.
33B.
34ROV.
106GM.
FJ183
104GM.
26PAD.
109GM.
FJ218
108GM.
75VF.
27PAD.
58RO.
99GM.
2PAD.
14GM.
35FP.
25B.
ICEMAN
29CIM.
101GM.
78VN.
110GM.
59S.
25S.
30MOCH
97GM.
TOR307
49GM.
TOR308
111GM.
112GM.
21ROV.

Figure S3

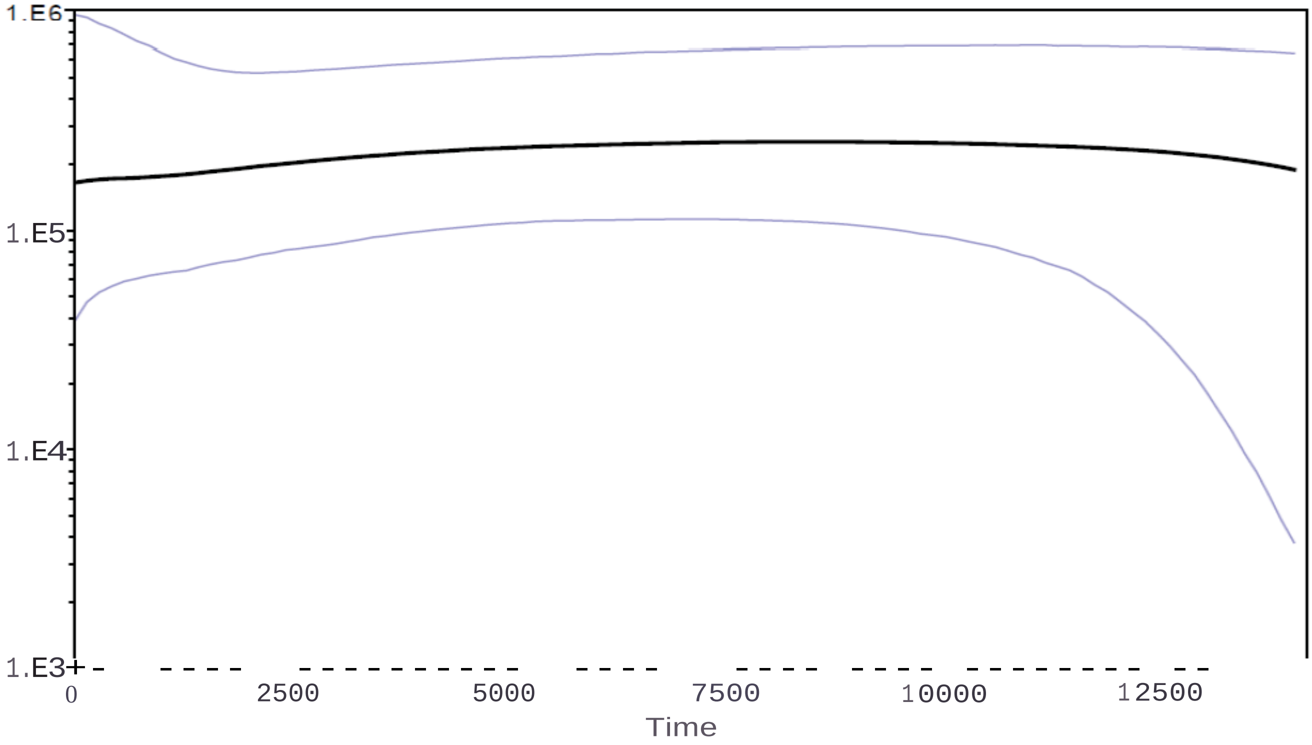

Supplement: Supplementary Information [file srep18932-s1.pdf]
